# Acetylsalicylic acid dosed at bedtime vs. dosed in the morning for circadian rhythm of blood pressure- a systematic review and meta-analysis

**DOI:** 10.3389/fcvm.2024.1346265

**Published:** 2024-10-22

**Authors:** Abdullah Nadeem, Taruba Rais, Minahil Aamir, Alexander Habte, Tasmiyah Siddiqui, Riyan Imtiaz Karamat, Rabbia Munsab, Ashna Habib

**Affiliations:** ^1^ Department of Medicine, Dow University of Health Sciences, Karachi, Pakistan; ^2^ Dow Medical College, Dow University of Health Sciences, Karachi, Pakistan; ^3^ Department of Surgery, Assab Military Hospital, Assab, Eritrea; ^4^ Department of Medicine, Rahbar Medical and Dental College, Lahore, Pakistan

**Keywords:** cardiovascular disease, aspirin, systolic blood pressure, diastolic blood pressure, hypertension, circadian rhythm, bedtime dosing

## Abstract

**Introduction:**

Cardiovascular disease (CVD) is a leading global cause of morbidity and mortality, with high systolic blood pressure (SBP) identified as a major risk factor. Aspirin (Acetylsalicylic acid—ASA) has been considered for CVD prevention, prompting questions about its optimal use in primary and secondary prevention and the ideal dosing time to maximize its impact on circadian blood pressure rhythms. Previous research suggests a potential benefit of bedtime aspirin dosing in reducing blood pressure, attributed to its effects on the renin-angiotensin-aldosterone system and nitric oxide production. This systematic review and meta-analysis aim to further explore the circadian effects of aspirin on blood pressure, focusing on the timing of administration.

**Methods:**

Adhering to PRISMA guidelines, a comprehensive search of PubMed, Cochrane Library, and clinicaltrials.gov was conducted. Randomized controlled trials (RCTs) involving patients aged >18 with cardiovascular history and hypertension were included. The primary objective was to assess the impact of bedtime-dosed and morning-dosed aspirin on systolic and diastolic blood pressure. Low-dose aspirin was administered for primary or secondary prevention. The Cochrane Risk of Bias tool evaluated study quality. Meta-analyses were conducted using RevMan 5.3, with mean deviations and 95% confidence intervals employed for outcomes.

**Results:**

Initial searches yielded 1,181 articles, with six studies meeting the inclusion criteria. These RCTs involved 1,470 patients, with 1,086 completing follow-up. Bedtime aspirin dosing demonstrated a significant reduction in both systolic and diastolic blood pressure compared to morning dosing (*p* < 0.05). Meta-analysis results for systolic blood pressure revealed a weighted mean difference of approximately 3.65 mmHg in favour of bedtime dosing, with low heterogeneity (*I*^2^ = 0%). For diastolic blood pressure, the weighted mean difference was 1.92, again favouring bedtime dosing, with 3% heterogeneity.

**Conclusion:**

This meta-analysis, involving over 1,300 cardiovascular/hypertensive patients, supports the effectiveness of bedtime aspirin in reducing systolic and diastolic blood pressure compared to morning dosing. The results align with previous findings but distinguish themselves by incorporating a more diverse patient population and addressing moderate heterogeneity. While the study's outcomes are promising, further research, including larger sample sizes and longer durations, is warranted for comprehensive clinical implementation. As the study exclusively focused on aspirin timing, future investigations should explore sustained blood pressure effects in patients with clinical indications for aspirin alongside other hypertensive medications.

## Graphical Abstract

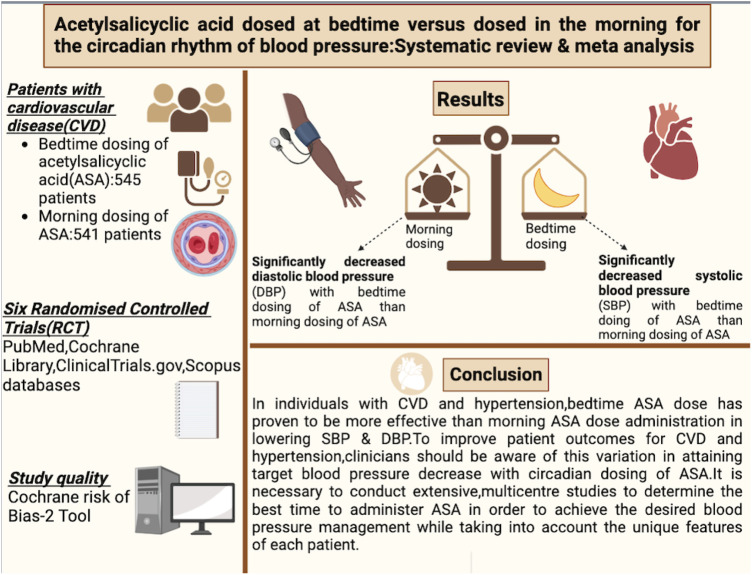

## 1 Introduction

Several diseases contribute to the makeup of the term cardiovascular disease (CVD). CVD is a major cause of death, morbidity, and healthcare resource expenditure in the world (1). CVD has a number of prognosis-determining modifiable risk factors. High systolic blood pressure (SBP) is attributed to be the leading risk factor for premature cardiovascular deaths. Globally in 2021, high SBP accounted for 10.8 million CVD-related deaths and 11.3 million overall. It is a particularly strong precipitator of ischemic heart disease, stroke, and intracerebral hemorrhage-related deaths (2). In the United States alone during the 2017–2020 period, 58.4% of adult non-Hispanic Black females had high blood pressure (HBP), the highest prevalence of all race and sex categories. The lowest prevalence is in Hispanic females at 35.3%. Of the HBP mortality population in 2020, 51.3% were females and 48.7% were males (3). Logically, HBP or High SBP tend to be impactful targets for the primary and secondary prevention of cardiovascular and cerebrovascular events in at-risk hypertensive patients. Aspirin or Acetylsalicylic acid (ASA) is one such drug attempting prophylaxis in this regard. Two questions arise for optimal aspirin clinical use. Firstly, whether it is more suited for primary or secondary prevention of CVD and secondly the ideal dose swallow time to cause the greatest beneficial impact on circadian rhythms of blood pressure in humans.

A previous meta-analysis answering the first question indicated that using aspirin for secondary prevention of CVD-related events yielded a greater absolute reduction in serious vascular events (6·7% aspirin vs. 8·2% control per year, *p* < 0.0001) as compared to the primary prevention group (0·51% vs. 0·57% per year, *p* = 0·0001). Additionally, there were increased major gastrointestinal and extracranial bleeds (0·10% vs. 0·07% per year, *p* < 0·0001) in the primary prevention patients on aspirin administration (4). Hence, for primary prevention patient populations the increased chance of bleeding events makes any rationale to use aspirin prophylactically questionable (4, 5).

Several factors such as serum nitric oxide, prostaglandin, angiotensin II, angiotensin-converting enzyme, renin-angiotensin-aldosterone system (RAAS) activity, expression of pro-inflammatory cytokines, and leukocyte adhesives contribute to HBP (6). Many of these influencers of HBP are known to be under the control of circadian clocks (7–9). The HBP-lowering effect of aspirin in the evening can be explained by the effect of inhibiting the otherwise increasing RAAS system activity, as well as production of the vasodilatory nitric oxide (10).

Normal individuals have a 10%–20% decrease in Blood Pressure (BP) at night. Non-dipping individuals with less than 10% dips in BP are linked to activation of the RAAS system (11) and at greater risk of CVD events (12, 13). This suggests the assumption that the night dipping phenomenon may neutralize the relatively higher blood pressures present in the day which are attributed to greater sympathetic system activity unmasking the full extent of aspirin BP lowering effects (14). It is also known that the antiplatelet action of aspirin is suboptimal during the morning while a significant reduction in platelet aggregation was obtained in a relevant randomized trial (15, 16). The multifactorial usefulness of aspirin in lowering BP via its effects on vascular endothelium, inhibiting thromboxane A2 mediated platelet aggregation paired with optimal bedtime administration deals a significant blow to HBP and as a result reoccurrences of CVD events such as Myocardial Infarction (MI) and stroke. Several clinical trials and previous meta-analyses support this evening/bedtime/night BP lowering hypothesis in CVD patients (15–17).

An issue with previous trials and meta-analyses on this subject is that the majority of the data was from populations of Chinese origin. Our updated meta-analysis includes RCTs conducted on populations other than Chinese origin. Additionally, we analyze only high-level evidence from RCTs, as reflected by our exclusion criteria.

This present analysis is intended to further establish the benefits of nocturnally dosed aspirin in countering the major modifiable risk factor of CVD, namely BP. If this quick fact-turning bedtime hypothesis keeping in mind individual patient BP circadian rhythms in mind is adopted widely enough the overall incidence of CVD and many of its various causative factors may visibly reduce worldwide.

## 2 Methods

### 2.1 Data sources and search strategy

This comprehensive review adheres to the meticulous reporting standards outlined in the Preferred Reporting Items for Systematic Review and Meta-Analysis (PRISMA) guidelines 2019 (18) discern studies meeting the criteria for inclusion, we methodically searched three databases, including PubMed, Cochrane Library, and clinicaltrials.gov. These searches were carried out autonomously by two of our study investigators and culminated on August 31, 2020. Our search keywords encompassed terms like “Aspirin,” “ASA,” “acetylsalicylic acid,” “Time,” “Morning,” “Night,” “Before Sleeping,” “Chronotherapy,” “Circadian rhythms,” “Cardiovascular,” “Hypertension,” combined with study-type identifiers such as “RCT,” “Randomized-Controlled trials,” and “trial.” Further details of our search strategy can be found in the Additional file (Supplementary Table S1).

### 2.2 Inclusion and exclusion criteria

(a) The study design included Randomized Control Trials (RCTs).
(b) The targeted population was patients aged >18 years with a history of cardiovascular and hypertension.
(c) The primary objective of our investigation was to evaluate the impact of bedtime-dosed and morning-dosed aspirin as an intervention and control, respectively, on systolic and diastolic blood pressure.
(d) Low-dose aspirin was administered for primary or secondary cardiovascular event prevention, either as a sole antiplatelet therapy or in combination with non-antiplatelet drugs, such as antihypertensive medications.
(e) Our study excluded abstracts, unpublished materials, reviews, book chapters, non-RCT studies, and studies published in languages other than English, as well as studies lacking clear reporting of primary and secondary outcomes.

### 2.3 Quality assessment and risk of bias in studies

The Cochrane Risk of Bias tool (19) was employed to evaluate the quality of the included RCTs. This assessment encompassed factors such as sequence generation, allocation concealment, blinding of participants and personnel, blinding of outcome assessment, handling of incomplete outcome data, selective outcome reporting, and other potential sources of bias. Each study was categorized as having a low, high, or unclear risk of bias for each of these factors. If a study was deemed to have a “high risk” of bias in any one category, it was classified as having an overall “high risk of bias”. Two independent assessors conducted the quality assessment, and any discrepancies were resolved through consensus or by consulting a third researcher.

### 2.4 Data extraction

Two investigators independently collected all pertinent data from the 7 included studies. This information, including general study characteristics (such as first authors, publication years, study type, and sample size), demographic details (covering age and gender), intervention and control specifics (including treatment frequency and duration), and outcome-related features (comprising outcome categories, definitions, and follow-up details), was systematically entered in Google sheets.

### 2.5 Data analysis

We conducted meta-analyses utilizing Cochrane's Review Manager (RevMan) version 5.3. Mean deviations with 95% confidence intervals (CIs) were employed to assess clinical outcomes, systolic and diastolic blood pressure, in patients with cardiovascular disease and hypertension who were undergoing Aspirin treatment at Morning and Bedtime respectively. We applied a fixed effects model to calculate combined effect estimates for comparing outcomes between the intervention and control groups. Heterogeneity was assessed using the *I*^2^ statistic, with a classification of significant heterogeneity (*I*^2^ ≥ 50%), interpreted based on study characteristics.

### 2.6 Ethical consideration

No ethical approval or patient consent was necessary for this meta-analysis since we solely utilized data extracted from RCTs identified in the literature search. All patient data used was entirely anonymized, eliminating the need for ethical clearance or individual patient consent.

## 3 Result

### 3.1 Literature search result

**Figure 1:**
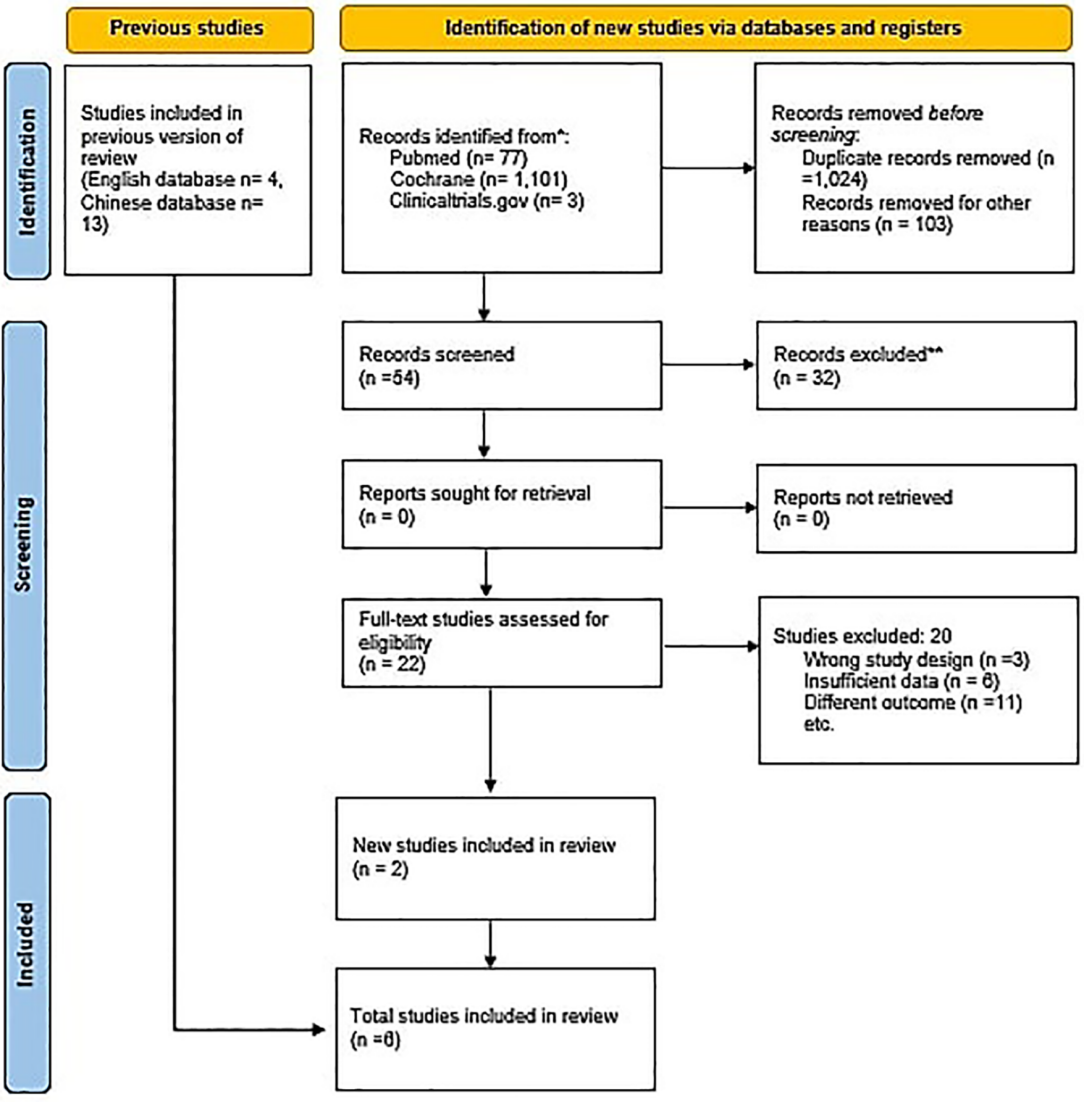
PRISMA flowchart. The PRISMA flow diagram for the systematic review detailing the database searches, the number of abstracts screened, and the full texts retrieved.

An initial search of the 3 databases i.e., PubMed, Cochrane and clinicaltrials.gov, revealed 1,181 potentially relevant articles, of which 157 remained after excluding duplicates. After applying eligibility criteria, we selected 6 articles for inclusion in the systematic review (20–25). The Preferred Reporting Items for Systematic Reviews and Meta-analyses flowchart shown in Figure 1 summarizes the literature search for this study. All included studies were given 100 mg of aspirin except Krasińska B, (25), in which 75 mg of acetylsalicylic acid was given to the participants.

### 3.2 Study characteristics and quality assessment

These trials randomly assigned a total of 1,470 patients, out of which 1,086 completed the follow-up with 545 in the intervention group (bedtime dose) and 541 in the control group (morning dose). Follow-up duration ranged from 12 weeks to 3 months. The baseline characteristics of all trials are summarized in Table 1.

**Table 1:** Basic characteristics of included studies.

Study	Year	Design	Population	Participants (*n*)	Sex,% female	Avg. BMI, kg/m^2^	Mean age, years	Intervention time, dose
Hermida et al. (20)	2005	Prospective, randomized, open-label, blinded end point (PROBE), parallel-group trial	Grade 1 (mild) essential hypertension	328	65.55	28.73	44.6	3 months, 100 mg
Hermida* et al. (21)	2005	Prospective, randomized, open-label, blinded end point (PROBE), parallel-group trial	Grade 1 (mild) essential hypertension	257	61.86	29	44.6	3 months, 100 mg
Hermida et al. (22)	2009	A single-center prospective, randomized, open-label, parallel-group, blinded endpoint (PROBE)	Prehypertension	244	56.55	28.13	42.9	3 months, 100 mg
Ayala and Hermida (23)	2010	Prospective, open-label, parallel-group, blinded endpoint (PROBE) trial	Grade 1 (mild) essential hypertension	316	58.9	28.65	44.1	12 weeks, 100 mg
Ji et al. (24)	2016	RCT	Grade 1-2 hypertension	150	49.33	-	62.4	3 months, 100 mg
Krasińska et al. (25)	2020	RCT	Coronary heart disease and hypertension	175	33.7	29.51	59.8	3 months, 75 mg

**Figure 2:**
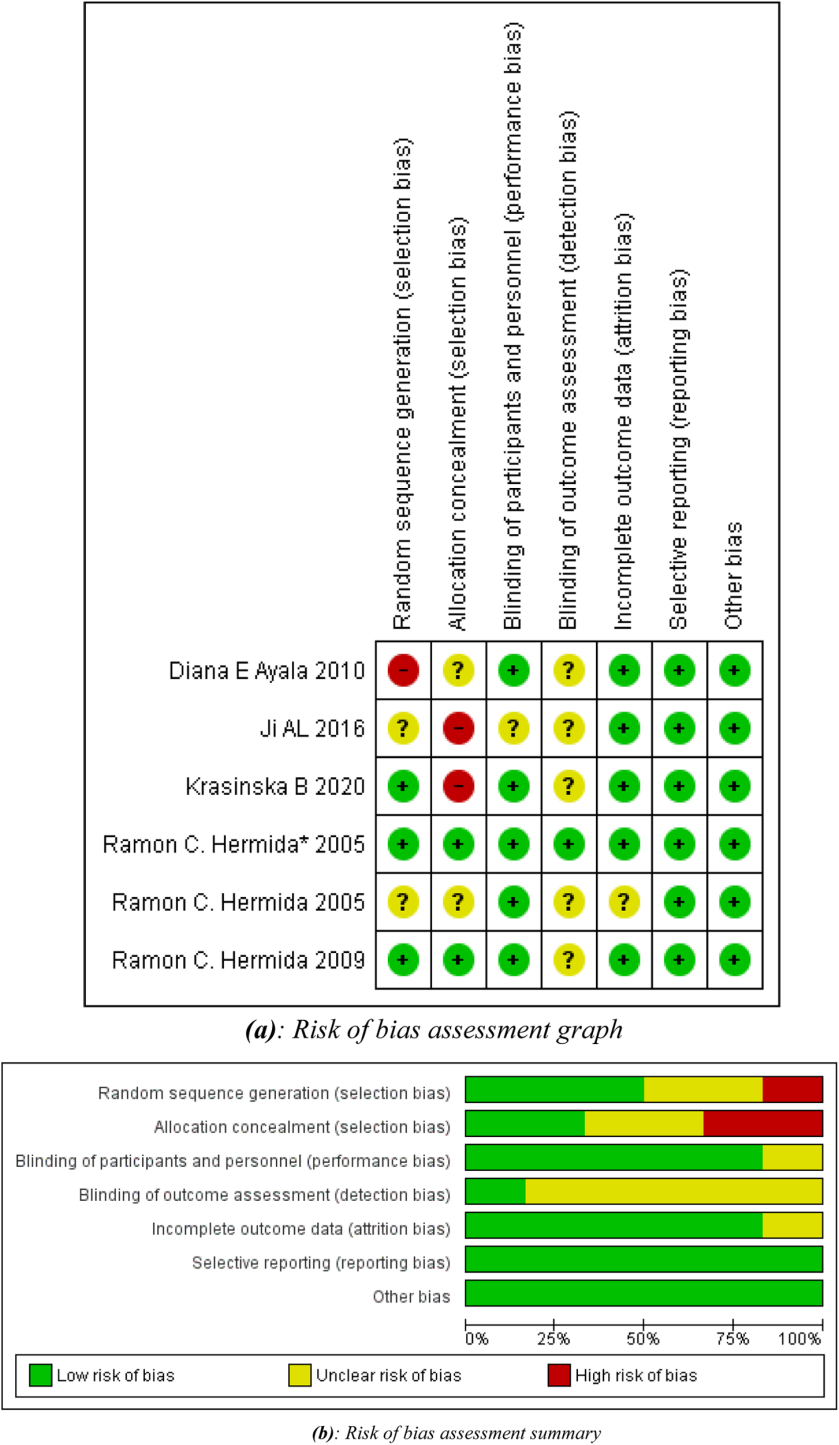
**(a)** Risk of bias assessment graph. **(b)** Risk of bias assessment summary.

All studies were of considerably high methodological quality as shown in Figures 2a,b. A detailed quality assessment of each study is given in Table 2.

**Table 2:** Quality assessment of included trials.

Trial	Sequence generation	Allocation concealment	Blinding	Detection bias	Attrition bias	Other bias
Hermida et al. (22)	Low	Low	Low	Unclear	Low	Low
Hermida et al. (20)	Unclear	Unclear	Low	Unclear	Unclear	Low
Hermida et al. (21)*	Low	Low	Low	Low	Low	Low
Ayala and Hermida (23)	High	Unclear	Low	unclear	Low	Low
Ji et al. (24)	Unclear	High	Unclear	Unclear	Low	Low
Krasińska et al. (25)	Low	High	Low	Unclear	Low	Low

### 3.3 Change in blood pressure in response to ASA chronotherapy

Seven distinct studies examined the impact of aspirin on blood pressure at various time intervals. The findings indicated that the administration of 75–100 mg of aspirin before bedtime resulted in a noteworthy reduction in both systolic and diastolic blood pressure when compared to morning dosing (both *p* < 0.05), signifying a significant difference in the outcomes.

#### 3.3.1 Meta-analysis results for systolic blood pressure

Systolic blood pressure represents the maximum force exerted by blood against arterial walls during each cardiac cycle, particularly during the heart's left ventricular contraction. Aspirin's anti-inflammatory properties enhance blood vessel flexibility, reducing resistance to blood flow, while its antiplatelet effects prevent clot formation, promoting improved circulation. These factors collectively contribute to a modest reduction in blood pressure, specifically systolic values.

**Figure 3:**
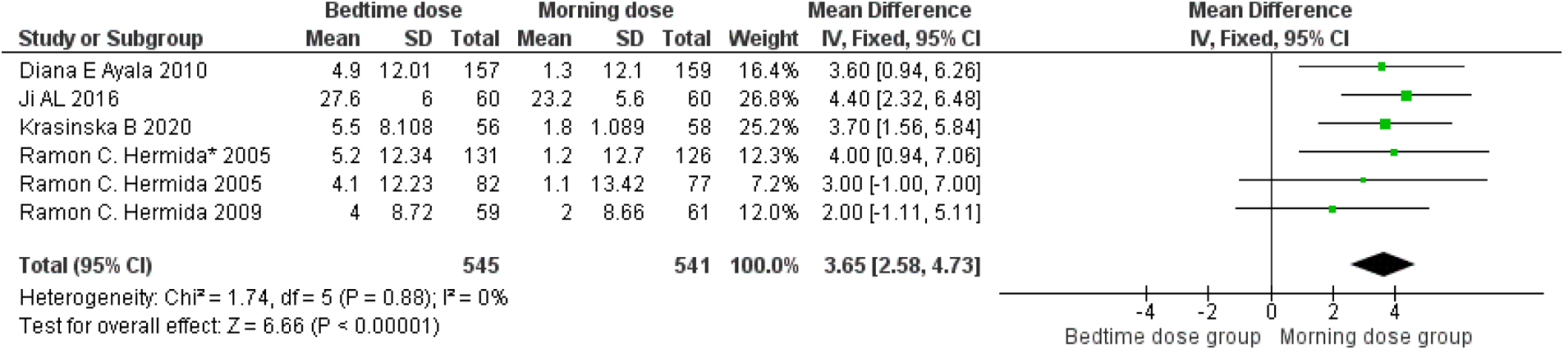
Bedtime vs. Morning dose of ASA on Systolic Blood Pressure (SBP). The diamond indicates the overall MD with an associated 95% CI. SD, standard deviation; IV, inverse variance; CI, confidence interval.

The analysis, depicted in Figure 3, underscores a more pronounced decrease in systolic blood pressure when aspirin is administered before bedtime. The data reveals a weighted mean difference (WMD) of approximately 3.65 (95 per cent confidence interval, 2.58–4.73) relative to morning dosing. Within the seven studies analyzed, a moderate level of heterogeneity at 0 per cent was observed, with a highly significant *p*-value of less than 0.00001, indicating substantial and statistically significant diversity in the results. Consequently, these findings substantiate the association between bedtime aspirin administration and a mean systolic blood pressure reduction of 3.65 mmHg in randomized controlled trials.

#### 3.3.2 Meta-analysis results for diastolic blood pressure

**Figure 4:**
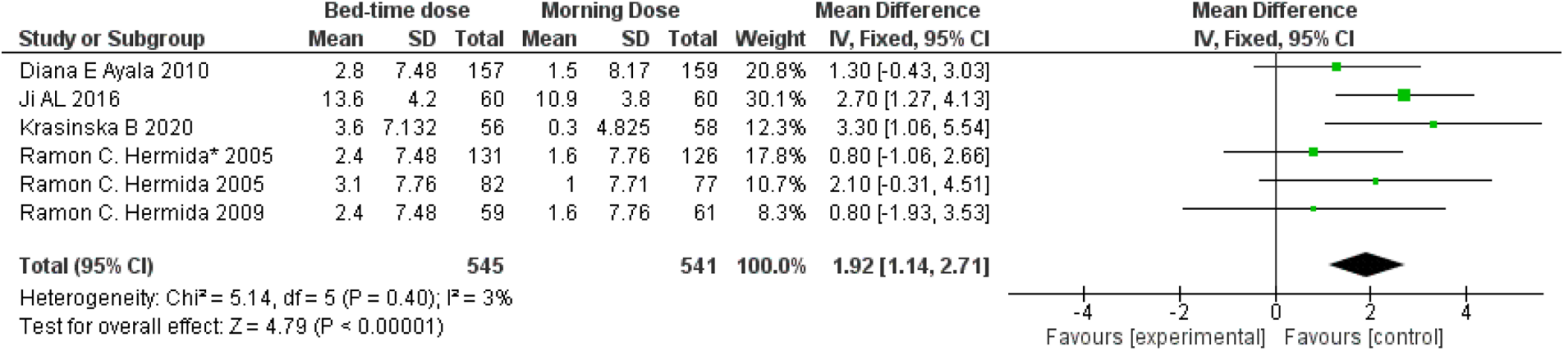
Bedtime vs. Morning dose of ASA on Diastolic Blood Pressure (DBP). The diamond indicates the overall MD with an associated 95% CI. SD, standard deviation; IV, inverse variance; CI, confidence interval.

Diastolic blood pressure, representing arterial pressure during the heart's resting phase, is influenced by aspirin's antiplatelet and anti-inflammatory properties, potentially enhancing blood vessel function. A comparative analysis illustrated in Figure 4 indicates a weighted mean difference of 1.92 (95 per cent confidence interval: 1.41–2.71) between individuals taking aspirin at bedtime and those taking it in the morning. This suggests a modest reduction in diastolic blood pressure for bedtime aspirin users. The observed 3 per cent heterogeneity, along with a remarkably low *p*-value of 0.00001, signifies statistically significant diversity in the studied populations, emphasizing the need for further exploration in this context.

## 4 Discussion

Our pooled analysis of six RCTs involving more than 1300 cardiovascular/hypertensive patient populations assessed the effects of different chronotherapy of aspirins over blood pressure and demonstrated that administration of aminosalicylic acid before bedtime resulted in a statistically significant reduction in systolic as well as diastolic blood pressure in comparison to the administration of same dosage of aspirin in the morning (Figures 3,4).

It is particularly noteworthy because this is the only meta-analysis that combined the results of RCTs with relatively moderate heterogeneity in contrast to other studies that reported their results with substantial heterogeneity (17) The findings of our study are consistent with the results reported by previous meta-analyses (17) that included 17 RCTs, however, there are many factors that make our study dissimilar from the previously published study. The prior meta-analysis consisted of 17 RCTs, out of which 13 were Chinese RCTs which could potentially create the bias of application of their reported findings only to the Chinese population. Apart from that, significantly high heterogeneity was reported in its results not only in total effect size but also in subgroup analysis. This substantially high heterogeneity with no resolution with subgroup analysis is another factor that distinguishes our study from previous meta-analyses. Thus, our systematic review and meta-analysis could serve as a source of robust findings owing to its moderate heterogeneity and diverse patient population. Nevertheless, the core outcomes of this and previous study share the same results of reduction in systolic blood pressure and diastolic blood pressure at evening dose rather than in the morning.

While our results show promise, questions linger about its practical implications in real-world clinical settings. The modest magnitude of this reduction prompts a critical consideration of its significance in the broader context of cardiovascular risk management. Thus, our study endeavours to bridge the gap between statistical significance and clinical applicability, aiming to investigate the true impact of bedtime dosing of aspirin on long-term cardiovascular health outcomes.

The probable explanation of such findings could be provided by the study that reported a significant reduction in renin activity of plasma for 24 h when aspirin is taken at bedtime in comparison to its administration in the morning. Furthermore, cortisol, dopamine and norepinephrine were also significantly reduced with their urinary excretion over a 24 h period proving a sound biological rationale for the drop in blood pressure at bedtime dosages of aspirin (26). Similarly, one of the multiple studies conducted by Hermida et al. on Chrono pharmacological effects of aspirin demonstrated the decline in blood pressure at bedtime based on the reason that bedtime-associated use of aspirin induces the nocturnal release of NO, reduces nocturnal peak of plasma renin activity, produced a favorable effect on vascular endothelial cells and blocks alpha- and beta-adrenergic receptors. However patient population (e.g., without co-morbidities) included in this study was very different from those that came for consulting in nephrology and cardiology (22). Although this study is strictly based on the established reporting specification of meta-analysis, we do not claim it to be implemented in clinical practice. While we observed significant outcomes, it is crucial to acknowledge the presence of moderate heterogeneity across the studies. This heterogeneity was effectively addressed through a sensitivity analysis, revealing that the study conducted by Maria V. Ruiz in 2019 was the primary source of maximum heterogeneity in both systolic and diastolic blood pressure. The rationale behind this heightened heterogeneity lies in the fact that the study encompassed a total of 20 primary centres. The intention-to-treat analyses were methodically conducted, comparing outcomes between bedtime and daytime intake periods. The analysis included adjustments for baseline values at the start of each period, utilizing mixed effects repeated measures analysis of covariance models.

Furthermore, the authors of the study extended their analytical models to account for potential confounding or modifying variables. This encompassed adjustments for changes in medication, age, sex, unhealthy habits, risk factors, comorbidities, and the time of study enrollment. This level of comprehensive adjustment was not reported in other trials, contributing to the observed heterogeneity.

In the vast field of medical research, our study stands out for its methodological rigour and clinical insight on aspirin chronotherapy. Although our results align with the previous meta-analysis, they are distinguished by their methodological refinement and the inclusion of diverse patient populations. It is within this context that our study gains significance and offers a nuanced exploration of the timing dynamics of aspirin administration in addition to a synthesis of existing evidence. Our pursuit represents a scholarly contribution aimed at adding valuable insights to the ongoing discussion on managing hypertension.

It is important to note that this meta-analysis has certain limitations. Firstly, the number of randomized controlled trials (RCTs) and the size of the study population were relatively small, which may limit the ability to draw consistent findings. Additionally, the total duration of dose administration was relatively short, which could potentially influence the accuracy of the results.

Certain studies included in this analysis exhibited some moderate bias, which should be taken into consideration when interpreting the results. This study exclusively compared data between individuals taking medicine in the morning and at bedtime, with no data available for those taking medicine in the morning and a placebo. Therefore, it is difficult to conclusively determine whether taking aspirin in the morning can effectively reduce blood pressure.

Before implementation of these findings in clinical practice, there's a need to conduct studies in future to evaluate the sustainability of blood pressure-reducing effects of bedtime aspirin in patients who carry clinical indications for intake of aspirin as they are on many other hypertensive medications as well which can likely dilute or interfere with the time-dependent impact of aspirin on blood pressure. However, if further, large and dedicated trials are conducted to explore the effects of dosage timing of aspirin on blood pressure keeping all the above-mentioned factors together, it could prove to be the most convenient way of improving control of blood pressure in patients who are already under the effect of so many therapeutic interventions since just a shift in the schedule of intake from morning to bedtime would be required to bring fine change in blood pressure in already debilitated patients, preventing the needs of further medicines and without effecting compliance of patients. In conclusion, further research is imperative to extract meaningful insights for clinical implementation. Additional studies with larger sample sizes, longer durations, and a more comprehensive approach are necessary in future to provide a more robust understanding of the effects of aspirin on blood pressure.

## 5 Conclusion

In conclusion, our systematic review and meta-analysis provide valuable insights into the impact of aspirin chronotherapy on blood pressure in cardiovascular and hypertensive patients. The analysis, based on high-quality randomized controlled trials (RCTs) with diverse populations, reveals a statistically significant reduction in both systolic and diastolic blood pressure when aspirin is administered at bedtime compared to morning dosing. This finding is consistent with previous studies but contributes unique strengths such as moderate heterogeneity and a more varied patient population.

The observed reduction in blood pressure is of clinical significance, considering the crucial role of elevated blood pressure as a major modifiable risk factor for cardiovascular diseases. The bedtime administration of aspirin demonstrates a potential strategy for optimizing the therapeutic effects of aspirin in countering hypertension, thereby reducing the risk of cardiovascular events. The underlying biological mechanisms, including the impact on plasma renin activity, cortisol, dopamine, and norepinephrine, offer a plausible rationale for the observed blood pressure-lowering effects during nighttime dosing.

However, it is essential to acknowledge the limitations of our analysis. The relatively small number of RCTs and study populations, along with the short duration of dose administration, warrant a cautious interpretation of the findings. Additionally, certain studies included in the analysis exhibited moderate bias, emphasizing the need for further dedicated trials.

Future research should focus on assessing the sustainability of bedtime aspirin's blood pressure-reducing effects, particularly in patients with clinical indications for aspirin who are concurrently on other hypertensive medications. Large-scale, long-term studies with a comprehensive approach are crucial for validating and extending the clinical implications of our findings. Before widespread implementation in clinical practice, further investigation is necessary to establish the optimal timing of aspirin administration for blood pressure control, considering patient compliance and potential interactions with other therapeutic interventions.

## Data Availability

The original contributions presented in the study are included in the article/Supplementary Material, further inquiries can be directed to the corresponding author.
