# Supplementary material for: Acetylsalicylic acid dosed at bedtime vs. dosed in the morning for circadian rhythm of blood pressure- a systematic review and meta-analysis
Source: Front Cardiovasc Med. 2024 Oct 22;11:1346265. doi: 10.3389/fcvm.2024.1346265 (PMC11536354; doi:10.3389/fcvm.2024.1346265)
Supplement: Supplementary file 1 [file Table1.pdf]

Supplementary Table 1: Detailed search strategy for each database

Database	String	Results
Pubmed	**coronary heart disease OR vascular disease) AND (blood pressure OR systolic blood pressure OR diastolic blood pressure)** ("aspirin"[MeSH Terms] OR "aspirin"[All Fields] OR "aspirins"[All Fields] OR "aspirin s"[All Fields] OR "aspirine"[All Fields] OR ("anal sci adv"[Journal] OR "asa"[All Fields]) OR ("aspirin"[MeSH Terms] OR "aspirin"[All Fields] OR ("acetylsalicylic"[All Fields] AND "acid"[All Fields]) OR "acetylsalicylic acid"[All Fields])) AND ("bedtime"[All Fields] OR "bedtimes"[All Fields] OR ("morning"[All Fields] OR "morning s"[All Fields] OR "mornings"[All Fields]) OR ("circadian rhythm"[MeSH Terms] OR ("circadian"[All Fields] AND "rhythm"[All Fields]) OR "circadian rhythm"[All Fields] OR ("circadian"[All Fields] AND "rhythms"[All Fields]) OR "circadian rhythms"[All Fields]) OR ("chronotherapy"[MeSH Terms] OR "chronotherapy"[All Fields] OR "chronotherapies"[All Fields]) OR "time-dependent"[All Fields]) AND ("cardiovascular system"[MeSH Terms] OR ("cardiovascular"[All Fields] AND "system"[All Fields]) OR "cardiovascular system"[All Fields] OR "cardiovascular"[All Fields] OR "cardiovasculars"[All Fields] OR ("hypertense"[All Fields] OR "hypertension"[MeSH Terms] OR "hypertension"[All Fields] OR "hypertension s"[All Fields] OR "hypertensions"[All Fields] OR "hypertensive"[All Fields] OR "hypertensive s"[All Fields] OR "hypertensives"[All Fields]) OR ("coronary disease"[MeSH Terms] OR ("coronary"[All Fields]	77

	AND "disease"[All Fields]) OR "coronary disease"[All Fields] OR ("coronary"[All Fields] AND "heart"[All Fields] AND "disease"[All Fields]) OR "coronary heart disease"[All Fields]) OR ("vascular diseases"[MeSH Terms] OR ("vascular"[All Fields] AND "diseases"[All Fields]) OR "vascular diseases"[All Fields] OR ("vascular"[All Fields] AND "disease"[All Fields]) OR "vascular disease"[All Fields])) AND ("blood pressure"[MeSH Terms] OR ("blood"[All Fields] AND "pressure"[All Fields]) OR "blood pressure"[All Fields] OR "blood pressure determination"[MeSH Terms] OR ("blood"[All Fields] AND "pressure"[All Fields] AND "determination"[All Fields]) OR "blood pressure determination"[All Fields] OR "arterial pressure"[MeSH Terms] OR ("arterial"[All Fields] AND "pressure"[All Fields]) OR "arterial pressure"[All Fields] OR ("blood pressure"[MeSH Terms] OR ("blood"[All Fields] AND "pressure"[All Fields]) OR "blood pressure"[All Fields] OR ("systolic"[All Fields] AND "blood"[All Fields] AND "pressure"[All Fields]) OR "systolic blood pressure"[All Fields]) OR ("blood pressure"[MeSH Terms] OR ("blood"[All Fields] AND "pressure"[All Fields]) OR "blood pressure"[All Fields] OR ("diastolic"[All Fields] AND "blood"[All Fields] AND "pressure"[All Fields]) OR "diastolic blood pressure"[All Fields]))	
Cochrane	(acetylsalicylic acid):ti,ab,kw AND (hypertension):ti,ab,kw AND (chronotherapy):ti,ab,kw OR ("circadian rhythm"):ti,ab,kw AND (blood pressure):ti,ab,kw	1,101
Clinicaltrials.gov	Condition/disease;<br>cardiovascular/ hypertension<br>Other terms;	3

	time-dependent OR chronotherapy<br>Intervention/treatment;<br>Acetylsalicylic acid	
